# Retraction: HPV16 E7-Dependent Transformation Activates NHE1 through a PKA-RhoA-Iinduced Inhibition of p38alpha

**DOI:** 10.1371/journal.pone.0218402

**Published:** 2019-06-10

**Authors:** The PLOS ONE Editors

Following publication of [1], the following concerns were noted:

- Figure 1A: E7 and GAPDH panels appear to contain background irregularities;
- Figure 1B: the bands in the total p38 and total JNK panels appear similar, cropped and adjusted for brightness and contrast;
- Figure 1C: total ERK1/2 panel contains vertical discontinuities;
- Figure 1C: GAPDH panel appears similar to the Figure 1B GAPDH panel 1B;
- Figure 2A: E7 panel has a vertical change in background between the nc and +tet lanes;
- Figure 2A: GAPDH panel background appears dissimilar between bands;
- Figure 4B: Phospho-p38 panel has a vertical change in background between the middle lanes;
- Figure 5A: Phospho RhoA and total RhoA panels have been heavily adjusted for brightness/contrast;
- Figure 6A: Active RhoA panel has vertical discontinuities between the 3 and 6 hr bands.

The corresponding author does not agree with the concerns raised and provided images in relation to Figure 1B totp38 and totJNK panels, Figure 1C total ERK panel, Figure 2A GAPDH and E7 panels, Figure 4B Phospho-p38 panel, Figure 5A phospho RhoA panel and Figure 6A Active RhoA panel, but these do not satisfactorily resolve the concerns raised for these items. The primary data underlying other figure panels has not been provided, which was attributed to the time that has passed since publication.

In light of the unresolved concerns that question the validity of the study’s findings, the *PLOS ONE* Editors retract the article.

RAC, MRG, PC, VC, MT, and SJR did not agree with retraction. GB, AB, RA, AC, SM, and AP did not respond.
